# Protective Effect of Vaccine Promoted Neutralizing Antibodies against the Intracellular Pathogen *Chlamydia trachomatis*

**DOI:** 10.3389/fimmu.2017.01652

**Published:** 2017-12-11

**Authors:** Anja Weinreich Olsen, Emma Kathrine Lorenzen, Ida Rosenkrands, Frank Follmann, Peter Andersen

**Affiliations:** ^1^Chlamydia Vaccine Research, Department of Infectious Disease Immunology, Statens Serum Institut, Copenhagen, Denmark

**Keywords:** chlamydia, vaccine, neutralizing antibodies, protection, cell-mediated immunity

## Abstract

There is an unmet need for a vaccine to control *Chlamydia trachomatis* (*C.t*.) infections. We have recently designed a multivalent heterologous immuno-repeat 1 (Hirep1) vaccine construct based on major outer membrane protein variable domain (VD) 4 regions from *C.t*. serovars (Svs) D–F. Hirep1 administered in the Cationic Adjuvant Formulation no. 1 (CAF01) promoted neutralizing antibodies in concert with CD4^+^ T cells and protected against genital infection. In the current study, we examined the protective role of the antibody (Ab) response in detail. Mice were vaccinated with either Hirep1 or a vaccine construct based on a homologous multivalent construct of extended VD4’s from SvF (extVD4^F^*4), adjuvanted in CAF01. Hirep1 and extVD4^F^*4 induced similar levels of Ab and cell-mediated immune responses but differed in the fine specificity of the B cell epitopes targeted in the VD4 region. Hirep1 induced a strong response toward a neutralizing epitope (LNPTIAG) and the importance of this epitope for neutralization was demonstrated by competitive inhibition with the corresponding peptide. Immunization with extVD4^F^*4 skewed the response to a non-neutralizing epitope slightly upstream in the sequence. Vaccination with Hirep1 as opposed to extVD4^F^*4 induced significant protection against infection in mice both in short- and long-term vaccination experiments, signifying a key role for Hirep1 neutralizing antibodies during protection against *C.t*. Finally, we show that passive immunization of Rag1 knockout mice with Hirep1 antibodies completely prevented the establishment of infection in 48% of the mice, demonstrating an isolated role for neutralizing antibodies in controlling infection. Our data emphasize the role of antibodies in early protection against *C.t*. and support the inclusion of neutralizing targets in chlamydia vaccines.

## Introduction

Worldwide, sexually transmitted infections (STIs) by *Chlamydia trachomatis* (*C.t*.) cause an annual estimated incidence of over 131 million cases (1). Despite effective antibiotics, the large proportion of asymptomatic infections (2) impedes the control of *C.t*. infections. In women, untreated infections can lead to severe long-term sequelae with pelvic inflammatory disease, chronic abdominal pain, ectopic pregnancy, and infertility as the most severe complications (3–5). The annual direct medical costs for chlamydial infections in the US alone exceed $500 million/year (6). Consequently, WHO has recently initiated a global roadmap targeting STIs. The long-term control strategy is to develop prophylactic vaccines (7).

Historically, the vast majority of vaccines work *via* the induction of antibodies (8). Antibody (Ab)-mediated neutralization can efficiently block pathogens from entering host cells or neutralize bacterial toxins. *C.t*. has a complex bi-phasic lifecycle and infects epithelial cells in a range of mucosal sites (9). The intracellular lifestyle of *C.t*. has resulted in a focus on cell-mediated immunity (CMI) for efficient recognition of infected cells and control through cytokines or cellular cytotoxicity (10–12). Optimally, a vaccine would completely prevent infection, but more likely, it will reduce the initial infectious load followed by CMI responses that will accelerate clearing of the remaining bacteria. A *C.t*. infection in itself may drive insufficient amounts of neutralizing antibodies to be protective. Instead, current thinking is that the infection also induces antibodies that have secondary protective functions as facilitators for both the adaptive and the innate immune responses (13, 14).

Our vaccine development strategy focused on the *C.t*. major outer membrane protein (MOMP). MOMP is the most prominent protein in the outer membrane and has been shown to function as a porin (15) and an adhesin (16). MOMP is the primary target for neutralizing antibodies during infection (16–18) and has four variable domains (VDs) protruding from the surface of *C.t*. (19). We recently described a recombinant engineered multivalent vaccine construct [heterologous immuno-repeat 1 (Hirep1)], based on extended variable domain 4 (extVD4) regions from the most prevalent serovars (Svs) D, E, and F. Formulated in the Cationic Adjuvant Formulation no. 1 (CAF01) adjuvant, this vaccine exposes key neutralizing epitopes in the VD4 domain together with conserved T cell epitopes (20). It can elicit broadly cross-reactive antibodies toward multiple serotypes. Furthermore, adoptive transfer of sera from vaccinated mice into recipient wild-type mice mediated protection against a primary vaginal challenge. Interestingly, depletion experiments showed that this protection was (at least partly) dependent upon CD4^+^ T cells suggesting that the mechanism behind was a synergy between neutralizing antibodies and CD4^+^ T cells (20). It is therefore still unclear what role these antibodies can play on their own in the protective immune response against *C.t*.

In the present study, we extend our studies on the Hirep1/CAF01 vaccine in the mouse model. We compare the immunogenicity and protective efficacy of Hirep1 with a similarly designed construct based on four repeats of extVD4 from SvF. We demonstrate that this construct has equal ability to induce CMI and Ab responses. However, despite a high degree of sequence similarity, it lacks the ability to induce neutralizing antibodies and protection. We also show that Hirep1-induced neutralizing antibodies can adoptively transfer protection into Rag1 knockout (KO) mice, which emphasizes the role of antibodies without the involvement of CMI responses in the early control of infection with *C.t*.

## Materials and Methods

### Organisms

The *C.t*. serovar D (*C.t*. SvD) (UW-3/Cx, ATCC VR-885), SvE (BOUR, VR-348B), and SvF (IC-Cal-3, ATCC VR-346) were purchased from the ATCC and propagated in HeLa-229 cells. Six-well plates were centrifuged at 750 *g* for 1 h at RT. *C.t*. elementary bodies (EBs) were harvested, purified, and quantified as described previously (21) and stored at −80°C in a sucrose-phosphate-glutamate (SPG) buffer. All procedures were done using Biosafety level 2 containments.

### Animals

Female B6C3F1 (C57BL/6J x C3H/HeN) mice, 6–8 weeks of age, were obtained from Harlan Laboratories. The mice were housed under standard environmental conditions and provided standard food and water *ad libitum*. Rag1-deficient (Rag1<tm1Mom>) mice (Rag1 KO) (22) were obtained from JAX Laboratories (JAX Stock #002216) and housed in high-barrier facilities at Statens Serum Institut. Animal experiments were conducted in accordance with regulations of the Danish Ministry of Justice and animal protection committees by Danish Animal Experiments Inspectorate Permit 2013-15-2934-00978 and in compliance with EU Directive 2010/63 and the US Association for Laboratory Animal Care recommendations for the care and use of laboratory animals.

### Fusion Protein Preparation and Peptide Synthesis

Recombinant proteins: Hirep1 and extended VD4’s from SvF (extVD4^F^*4) were produced as follows: based on the amino acid sequences (NCBI–GenBank) with an added N-terminal histidine tag, synthetic DNA constructs were codon-optimized for expression in *Escherichia coli* followed by insertion into the pJexpress 411 vector (DNA2.0). To avoid disulfide bridge formation during recombinant production, all cysteines were exchanged with serines. Purification was done essentially as described in Ref. (23). The 9-mer biotinylated pepset was produced by Mimotopes (United Kingdom) and the 20-mer peptides were produced by GeneCust (Luxembourg). For amino acid (aa) sequences see Ref. (20) and Table 1.

**Table 1:** Sequences of the VD4-based vaccine constructs.

Constructs	Extended VD4 sequences
extVD4^D^	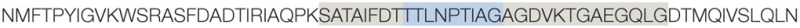

extVD4^E^	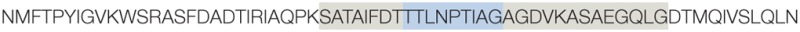

extVD4^F^	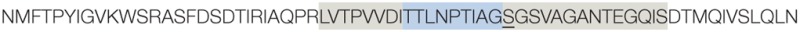

Hirep1	extVD4^D^-extVD4^E^-extVD4^F^

extVD4^F^*4	extVD4^F^-extVD4^F^-extVD4^F^-extVD4^F^

Overview and sequences of the extended VD4-based constructs Hirep1 and extVD4^F^*4 used in the present study. Gray box: VD4 region, Blue box: conserved sequence within the VD4 region. In the SvF sequence, the cysteine has been replaced with a serine.

### Mouse Immunization and Infection

Mice were immunized with 5 μg/dose/route of recombinant immunorepeat (extVD4^F^*4 or Hirep1) constructs. Mice received a total of three immunizations at 2-week intervals either subcutaneously (SC) at the base of the tail in a total volume of 200 µl or simultaneously with the intranasal (IN) route in a volume of 30 µl. The antigens were diluted in Tris-buffer (pH 7.4) and mixed by vortexing 1:1 with 100 µl (SC) or 15 µl (IN) CAF01 adjuvant consisting of 50 μg/dose of the glycolipid trehalose 6,6′-dibehenate (TDB) incorporated into 250 μg/dose of cationic liposomes composed of dimethyldioctadecyl-ammonium. The mice were rested for 6 weeks (short-term) or 72 weeks (long-term). Ten and 3 days before *C.t*. SvD or SvF challenge, the estrus cycle was synchronized by injection of 2.5 mg Medroxyprogesteronacetat (Depo-Provera; Pfizer) increasing mouse susceptibility to chlamydial infection by prolonging diestrus (24). The mice were challenged intravaginally (i.vag.) with 4 × 10^4^ *C.t*. SvD (Rag1 KO mice), 1 × 10^6^ inclusion forming unit (IFU) of *C.t*. SvF (B6C3F1 mice, short-term experiment), and 4 × 10^3^ IFU of *C.t*. SvF (B6C3F1 mice, long-term experiment) in 10 µl SPG buffer.

### Measurement of Ab Levels in Plasma/Serum and Swab Material

Blood was collected 10 days after last vaccination for quantification of vaccine-specific antibodies by enzyme-linked immunosorbent assay (ELISA). For isolation of serum, the tubes were centrifuged for 10 min at 10,000 *g*. To separate plasma, samples were centrifuged 10 min at 500 *g*. Maxisorp Plates (Nunc, Denmark) were coated with either recombinant antigens (1 µg/ml) or live *C.t*. SvD–F (5 µg/ml). The plasma or serum samples were diluted 1:20 and fivefold serially diluted before being added to coated maxisorp plates.

Enzyme-linked immunosorbent assay reactivity against the 9-mer overlapping biotinylated peptide panels spanning the extVD4 region of SvD, SvE, and SvF was investigated. Briefly, precoated ELISA plates were obtained from Mimotopes, blocked with skimmed-milk powder, washed and incubated with plasma prediluted 1:200. Swab material was collected at PID3 in 600 µl SPG buffer and added undiluted to coated plates. Antigen-specific total IgG was detected with isotype-specific HRP-conjugated rabbit antimouse (Zymed). The substrate was TMB-PLUS (Kem-En-TEC, Denmark). Endpoint titers were calculated as the highest dilution where OD_450–620_ exceeds the cutoff value. Cutoff values were calculated for each dilution step as mean OD_450–620_ of naive mice + 3 × SD_(naive mice)_.

### Chlamydia-Specific Cellular Responses

Splenocytes (four individual mice/group) were stimulated for 1 h with 5 µg/ml of Hirep1 or extVD4^F^*4 at 37°C/5% CO_2_ and subsequently incubated for 5 h at 37°C with 10 µg/ml brefeldin A (Sigma-Aldrich, USA) at 37°C. The intracellular cytokine staining procedure was done essentially as described in Ref. (25). The following antibodies were used for surface and intracellular staining: FITC anti-CD8a (53–6.7), APC-eF780-anti-CD4 (GK1.5), PE-anti-tumor necrosis factor-alpha (TNF-α), APC-anti-interleukin (IL)-2, PE-Cy7-anti-interferon gamma (IFN-γ), and PerCP-Cy5.5-anti-IL17. All antibodies were purchased from BD Pharmingen or eBiosciences. Responses were analyzed using a FACSCanto flow cytometer (BD) and FlowJo v.10.2 (Tree Star Inc.). Blood lymphocytes (PBMC’s) were purified on a density gradient. Cells were pooled from eight mice and cultured in duplicate or triplicate in round-bottom plates (Nunc, Denmark) containing 2 × 10^5^ cells/well in a volume of 200 µl RPMI-1640 supplemented with 5 × 10^−5^ M 2-mercaptoethanol, 1 mM glutamine, 1% pyruvate, 1% penicillin–streptomycin, 1% HEPES, and 10% fetal calf serum (FCS) (Invitrogen, Denmark). Three pools of eight mice were analyzed per group. The cells were re-stimulated with overlapping 20-mer peptides (2 µg/ml) covering the extVD4 region from SvD, E, and F [for aa sequences see Table 1 and Ref. (20)]. As a positive control for cell viability and as a negative control, cells were stimulated with Concanavalin A (5 µg/ml) and media, respectively. After 72 h of incubation at 37°C in 5% CO_2_, supernatants were harvested and stored at −20°C before use. The amounts of secreted IFN-γ were determined in supernatants by ELISA as previously described (26).

### Neutralization

Blood samples were collected 3 weeks post last vaccination and sera were isolated. The neutralization assay was performed essentially as described in Ref. (27). Briefly, HaK cells (ATCC) were grown to confluence in 96-well flat-bottom microtiter plates. The adherent cells were maintained in RPMI-1640 (Gibco BRL) with 5 × 10^−5^ M 2-mercaptoethanol, 1 mM glutamine, 1% pyruvate, 10 µg/mL gentamicin, and 5% heat-inactivated FBS at 37°C/5% CO2. The *C.t*. stocks were diluted and mixed 1:1 with a heat-inactivated, serial diluted serum pool (*n* = 16 mice/group). The suspension was inoculated onto HaK cells in duplicates and incubated for 24 h. In the competitive neutralization inhibition assay, Hirep1 sera were preincubated with 20 µg/ml of peptides prior to the addition of SvD. Inclusions were visualized by staining with polyclonal rabbit antirecombinant CT043 serum, followed by Alexa 488-conjugated goat antirabbit immunoglobulin (Life Technologies). Cell staining was done with Propidium Iodide (Invitrogen). The results were calculated as the percentage reduction in mean IFU relative to control sera. A 50% or greater reduction in IFU relative to the control was defined as neutralizing.

### Passive Transfer of Immune Serum to Rag1 KO Mice

Two experiments were done. Sera were isolated from B6C3F1 mice previously vaccinated three times SC with 5 µg Hirep1/CAF01. In both experiments, mice vaccinated with CAF01 alone were included. The sera were heat-inactivated, sterile filtered and transferred by the intravenous and intraperitoneal routes to 10 (Exp.1) or 15 (Exp. 2) Rag1 KO mice using an equivalent of 2.7 donor:1 recipient. Rag1 KO mice receiving serum from control mice were used for comparison. Three days post serum transfer the mice were challenged with 4 × 10^4^ IFU/mouse of *C.t*. SvD and swabbed, as described under “Vaginal *C.t*. load.” The results are shown both as individual experiments (Figure S1 in Supplementary Material) and as a pool of the two experiments (Figure 5).

### Vaginal *C.t*. Load

Vaginal swabs were obtained at 3, 7, 10, 14, 17, or 21 days postinfection. Swabs were vortexed with glass-beads in 0.6 ml SPG buffer and stored at −80°C until analysis. The infectious load was assessed as described in Ref. (28). Inclusions were visualized by staining with polyclonal rabbit anti-MOMP serum made in our laboratory, followed by an Alexa 488-conjugated goat antirabbit immunoglobulin (Life Technologies, Denmark). Background staining was done with propidium iodide (Invitrogen, Denmark). IFUs were enumerated by fluorescence microscopy observing at least 20 individual fields of vision for each well. Culture-negative mice were assigned the lower cutoff of 4 IFU.

### Histopathology

Mice were anesthetized and euthanized by cervical dislocation and necropsied at PID21. The entire reproductive tract was removed en bloc and fixed in formalin. After fixation, the tissue was processed and embedded in paraffin according to standard procedures. Sections were cut to include the uterine horns, oviducts, and ovaries in the same section. The sections were mounted on Superfrost glass and stained with hematoxylin and eosin. Slides were scanned with ×20 objective and at 1,360 × 1,024 resolution by the 3D Histech Pannoramic Midi scanner (3D HISTECH Ltd., Budapest, Hungary) and evaluated with CaseViewer software (3D HISTECH Ltd., Budapest, Hungary) (Nordic Biosite). The genital tract tissue was evaluated for the degree of inflammatory infiltrates/presence of inflammatory cells according to the following semi-quantitative scoring system: (0) No cellular infiltration. (1) Infiltration at a single focus or scattered single cells. (2) Infiltration at two to four foci, small/moderate accumulations or confluent infiltration. (3) Infiltration at numerous (>4) foci/aggregates or moderate confluent infiltration. (4) Severe/extreme confluent infiltration. The sections were blinded to the treatment group and assessed by a pathologist.

### Statistical Analysis

GraphPad Prism 7 was used for data handling, analysis, and graphic representation. The differences in log 10 IFU obtained in the efficacy studies were analyzed using Kruskal–Wallis test followed by Dunn’s multiple comparison tests or the Mann–Whitney. Fisher’s exact test compared numbers of cleared and non-cleared Rag1 KO mice. Ab titers and pathology scoring were analyzed by the Mann–Whitney test. *P* < 0.05 was considered significant.

## Results

### Immunogenicity of extVD4^F^*4 in Comparison with Hirep1

**Figure 1:**
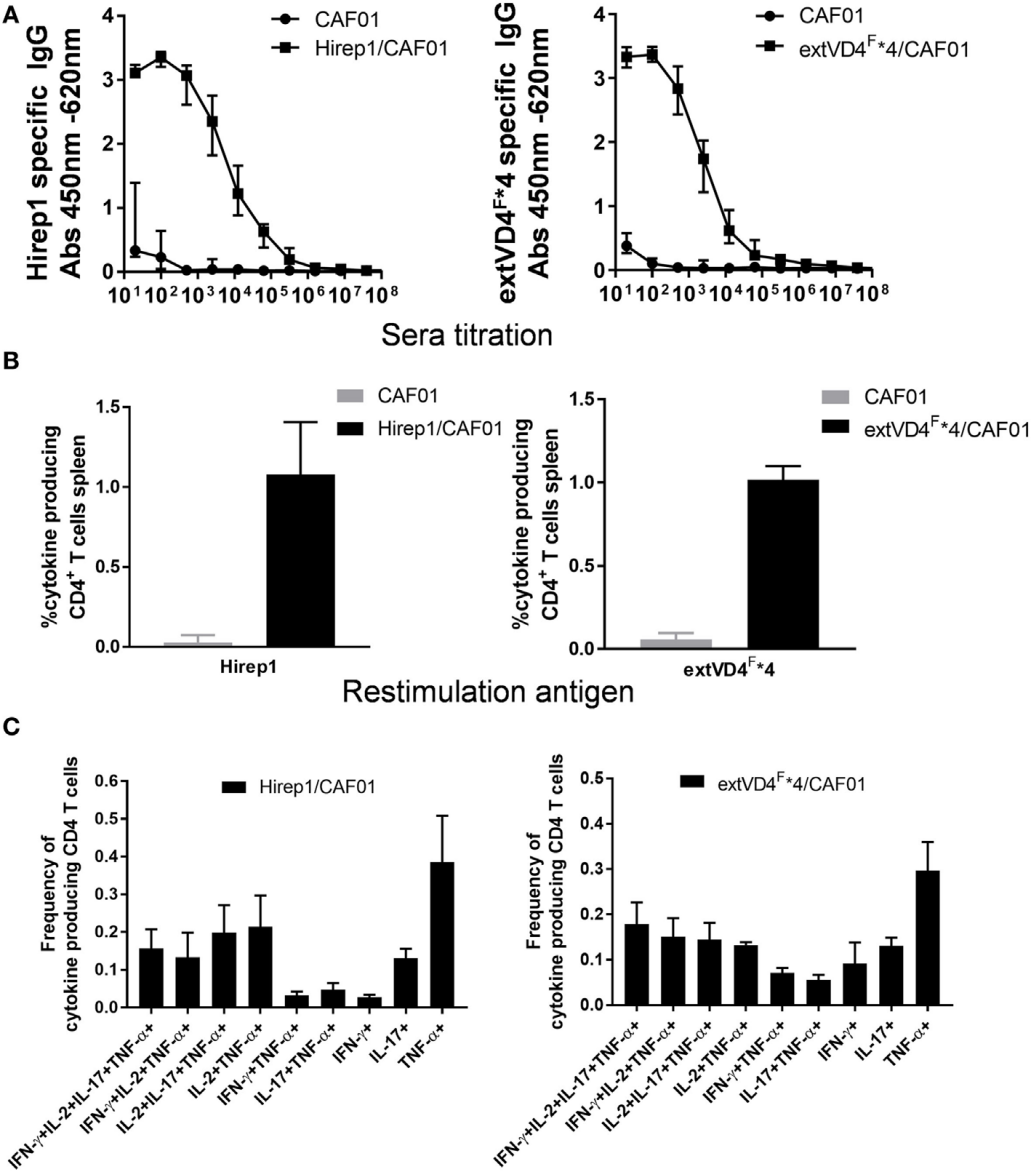
Vaccine-induced antibody (Ab) and cell-mediated immunity (CMI) response. **(A)** Plasma samples (*n* = 16) were isolated 1-week post third vaccination, serially diluted, and added to heterologous immuno-repeat 1 (Hirep1)- or extended VD4’s from serovar F (extVD4^F^*4)-coated plates, and antigen-specific IgG was analyzed by enzyme-linked immunosorbent assay. Each dot represents the median OD value ± interquartile range at each titration step. **(B)** The percentage of cytokine producing CD4^+^ T cells was measured by flow cytometry in splenocytes 3 weeks after last vaccination. CD4^+^ T cells that after restimulation with either Hirep1 or extVD4^F^*4 produced at least one of the cytokines, interferon gamma (IFN-γ), tumor necrosis factor-alpha (TNF-α), interleukin (IL)-2, or IL-17 were scored as cytokine positive CD4^+^ T cell. Cells producing more than one cytokine was only included once in the analysis. Each bar represents the mean level ± SEM of four individual mice. **(C)** The same spleen cells were used to investigate the polyfunctionality of the vaccine-specific T cells by measuring the frequencies of CD4^+^ T cells producing any combination of IFN-γ, TNF-α, IL-2, and IL-17. Only cytokine expression combinations with frequencies above 0.05% in at least one of the two groups were included in the figure. Results are shown for one of the two independent experiments.

Despite a high degree of sequence similarities, we have previously observed that the VD4 regions from SvE and SvF (extVD4^E^ and extVD4^F^, Table 1) generate immune responses with different capacity to neutralize *C.t*. (20). With the purpose of directly comparing the protective efficacy of two similarly designed constructs with different capacity to induce neutralizing antibodies, we designed an immunorepeat based on four repeats of the extVD4^F^ region–extVD4^F^*4 (Table 1) and compared it directly with Hirep1. B6C3F1 mice were vaccinated three times with Hirep1 or extVD4^F^*4 adjuvanted with CAF01. Mice were immunized simultaneously *via* the IN and SC routes, a vaccination regime previously demonstrated to be effective in inducing combined systemic and mucosal immune responses (20, 29). After vaccination, antigen-specific Ab, and CMI responses were compared (Figure 1). Hirep1 and extVD4^F^*4 both induced a high level of vaccine-specific IgG antibodies in plasma with endpoint titers of 312,500 and 62,500, respectively (Figure 1A). The CMI responses were measured by FACS analysis on total numbers of cytokine (IFN-γ, IL-17, TNF-α, IL-2) producing CD4^+^ T cells and demonstrated around 1% cytokine positive CD4^+^ T cell induced by both constructs (Figure 1B). In both groups, the functionality of the CD4^+^ T cell population was diverse and consisted preferentially of CD4^+^ T cells producing IL-2 in various combinations (Figure 1C). This is in agreement with published results showing that CAF01 primes memory responses with capacity for IL-2 production (30).

### Ab Neutralizing Capacity and Bacterial Surface Recognition

**Figure 2:**
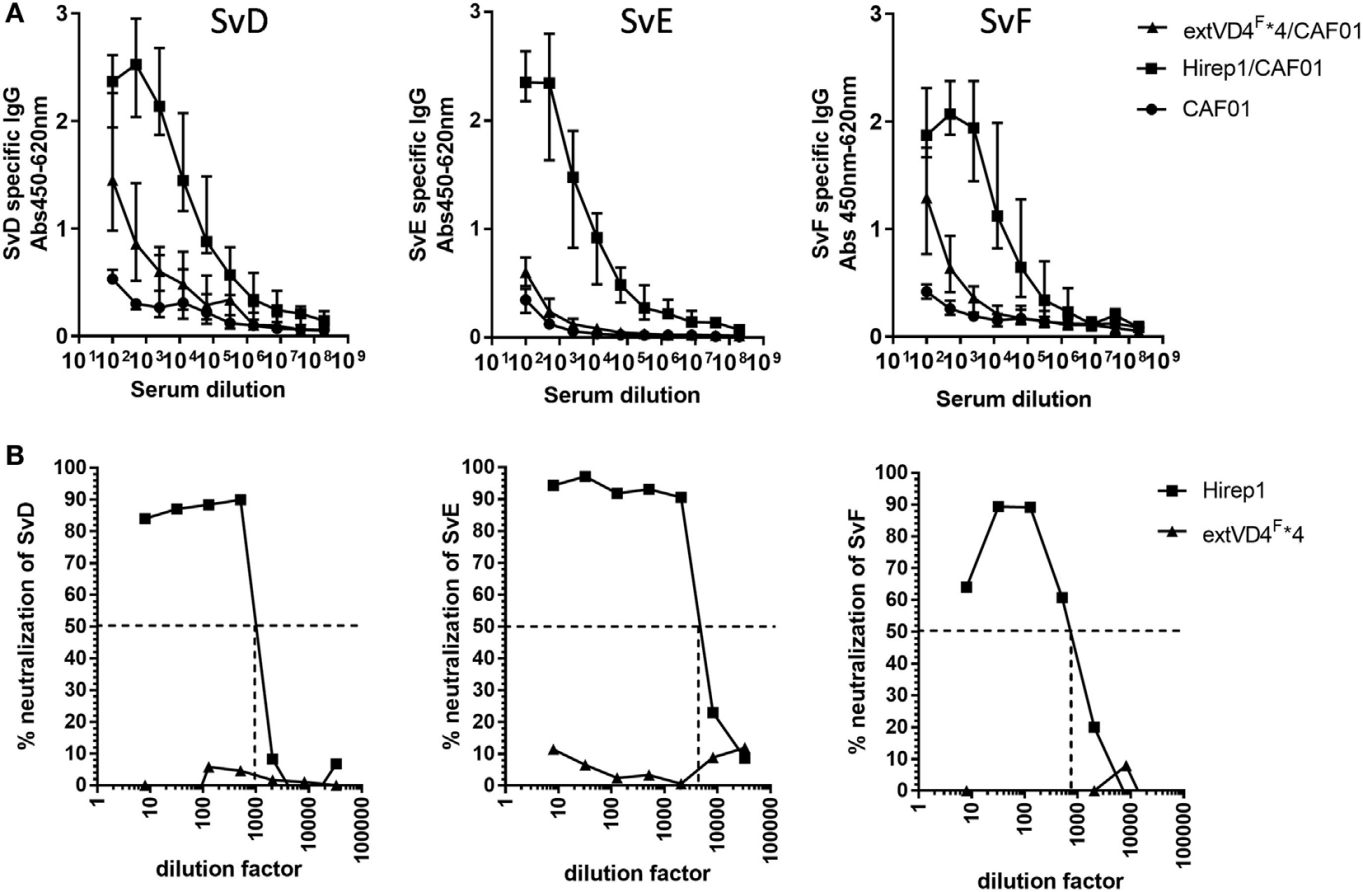
Elementary body (EB) Surface recognition and neutralizing capacity of the VD4 vaccines. **(A)** Plasma samples (*n* = 16) were serially diluted and added to Chlamydia trachomatis serovar (Sv) D-, SvE-, and SvF-coated plates and antigen-specific IgG was analyzed by enzyme-linked immunosorbent assay. Each dot represents the median OD value ± interquartile range at each titration step. **(B)** *In vitro* neutralization of SvD, SvE, and SvF. Sera pooled for each group (*n* = 16) were titrated, mixed with a fixed concentration of bacteria, inoculated onto a HaK cell monolayer, fixed and inclusions counted. The dotted line indicates the reciprocal 50% neutralization titer.

Having demonstrated similar levels of immunogenicity at both the CMI and Ab levels, we proceeded to investigate and compare the functional effect of the antibodies, i.e., their capacity to recognize the bacterial surface and to induce neutralization. Hirep1-specific antibodies had a broad Sv specificity and strongly recognized the surface of both Svs D, E, and F, whereas serum from extVD4^F^*4 vaccinated mice only demonstrated a very weak surface recognition (Figure 2A). The capacity to bind to the bacterial surface correlated strongly with the functional activity of the antibodies measured in a complement-independent *in vitro* neutralizing assay (Figure 2B). Hirep1 generated sera neutralized SvD–F with reciprocal 50% neutralizing titers ranging from 750 to 4,000, whereas the serum generated after extVD4^F^*4 vaccination was unable to induce neutralization of any of the three Svs tested.

### Specificities of Ab and CMI Responses after Hirep1 and extVD4^F^*4 Vaccination

**Figure 3:**
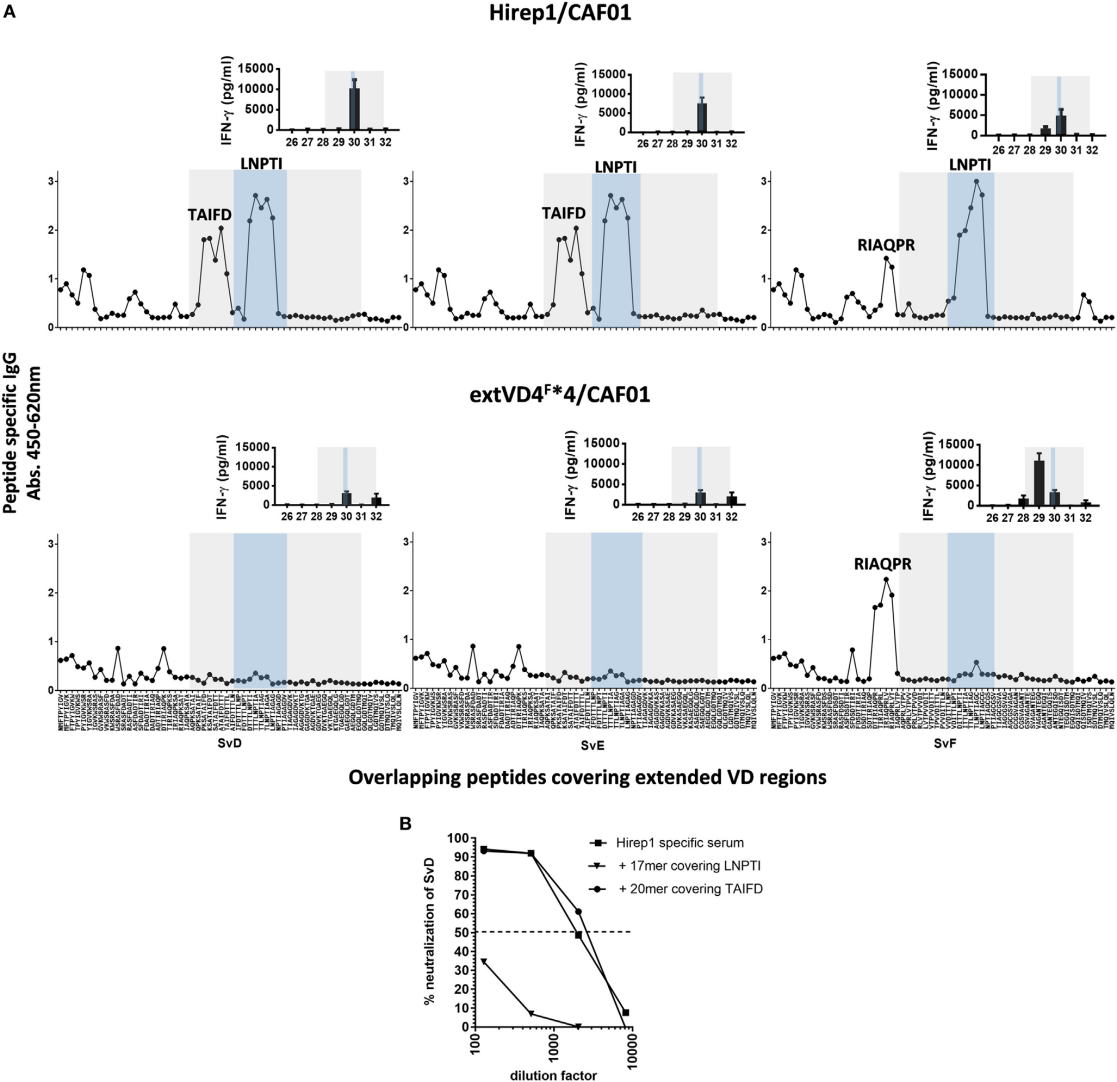
Fine specificity of antibody (Ab) and T cell responses after vaccination. **(A)** B6C3F1 mice were immunized with heterologous immuno-repeat 1 (Hirep1) or extended VD4’s from serovar F (extVD4^F^*4) (*n* = 16). After vaccination sera from immunized mice were pooled, diluted 1:200 and the fine specificity of the IgG Ab response were studied using a panel of biotinylated overlapping peptides (9-mers with 8AA overlap) representing the extVD4 region from serovar (Sv) D, SvE, and SvF. Inserts: VD4-specific interferon gamma (IFN-γ) responses were studied using panels of 20-mer peptides with 10aa overlap spanning the extVD4 regions from SvD, SvE, and SvF. Each bar represents mean of 8–9 individual wells ± SEM. The 8–9 individual wells came from duplicate or triplicate culturing of three pools each representing pools of PBMC’s from eight mice. The Ab and T cell responses identified within the VD4 region and within the conserved sequence in the VD4 region are depicted with the gray and blue boxes, respectively (see also Table 1). **(B)** Competitive peptide inhibition of *in vitro* neutralization of *C.t*. SvD with two peptides representing identified B cell epitope regions in Hirep1.

Given the pronounced difference in the functional effect of the Ab response promoted by the two constructs, we next analyzed the fine specificity of the responses. Ab specificity was determined by the reactivity against sequential and overlapping nonapeptides covering the extended VD4 regions of SvD, E, and F (Figure 3A). Abs from Hirep1 vaccinated mice induced a broad immune response with a very strong recognition of peptides spanning the highly conserved TTLNPTIAG region (blue box) with amino acids LNPTI as a critical binding motif. In addition, Hirep1 vaccinated mice recognized a SvD/E-specific sequence covering aa TAIFD and the SvF-specific sequence covering aa RIAQPR. In contrast, the Ab response generated by the SvF immunorepeat differed markedly with a very strong and focused response toward the SvF-specific sequence covering amino acids RIAQPR. This response did not include the highly conserved TTLNPTIAG region.

Epitope-specific T cell responses were determined by measuring the *in vitro* stimulatory properties of overlapping 20-mer peptides (10aa overlap) on PBMC’s from vaccinated mice. After stimulation, IFN-γ release was measured by ELISA (Figure 3A, inserts). The SvF-specific P29 and P30 from SvD, E, and F were the dominant T cell epitope regions recognized after vaccination with both constructs. However, whereas P30 is the dominant T cell epitope in Hirep1 vaccinated mice, SvF P29 dominate the CMI response in extVD4^F^*4 vaccinated mice—overlapping with the regions inducing the strongest Ab response. Having demonstrated that Hirep1 in contrast to extVD4^F^*4-specific serum was able to neutralize *C.t*., we continued by investigating the relative importance of the two major epitopes unique for Hirep1 as targets for neutralization; the TAIFD and the highly conserved LNPTI regions. To assess their relative role in neutralization, we used a competition neutralization assay with Hirep1 serum (Figure 3B). A 17-mer peptide FDTTT**LNPTI**AGAGDVK covering the LNPTI region and an overlapping 20-mer peptide RIAQPKSA**TAIFD**TTTLNPT (SvD/E P29) covering the SvD/E-specific TAIFD region were pre-incubated with serum from Hirep1 immunized mice and the mixture was further incubated for 45 min. with a fixed concentration of SvD before being transferred to HAK cells. Pre-incubation with the 17-mer peptide covering the LNPTI sequence completely abrogated the neutralization capacity, whereas blocking the serum with the peptide spanning the SvD/E-specific region TAIFD had no effect on the neutralizing capacity of the serum.

### Short- and Long-term Protection against *C.t*. SvF

**Figure 4:**
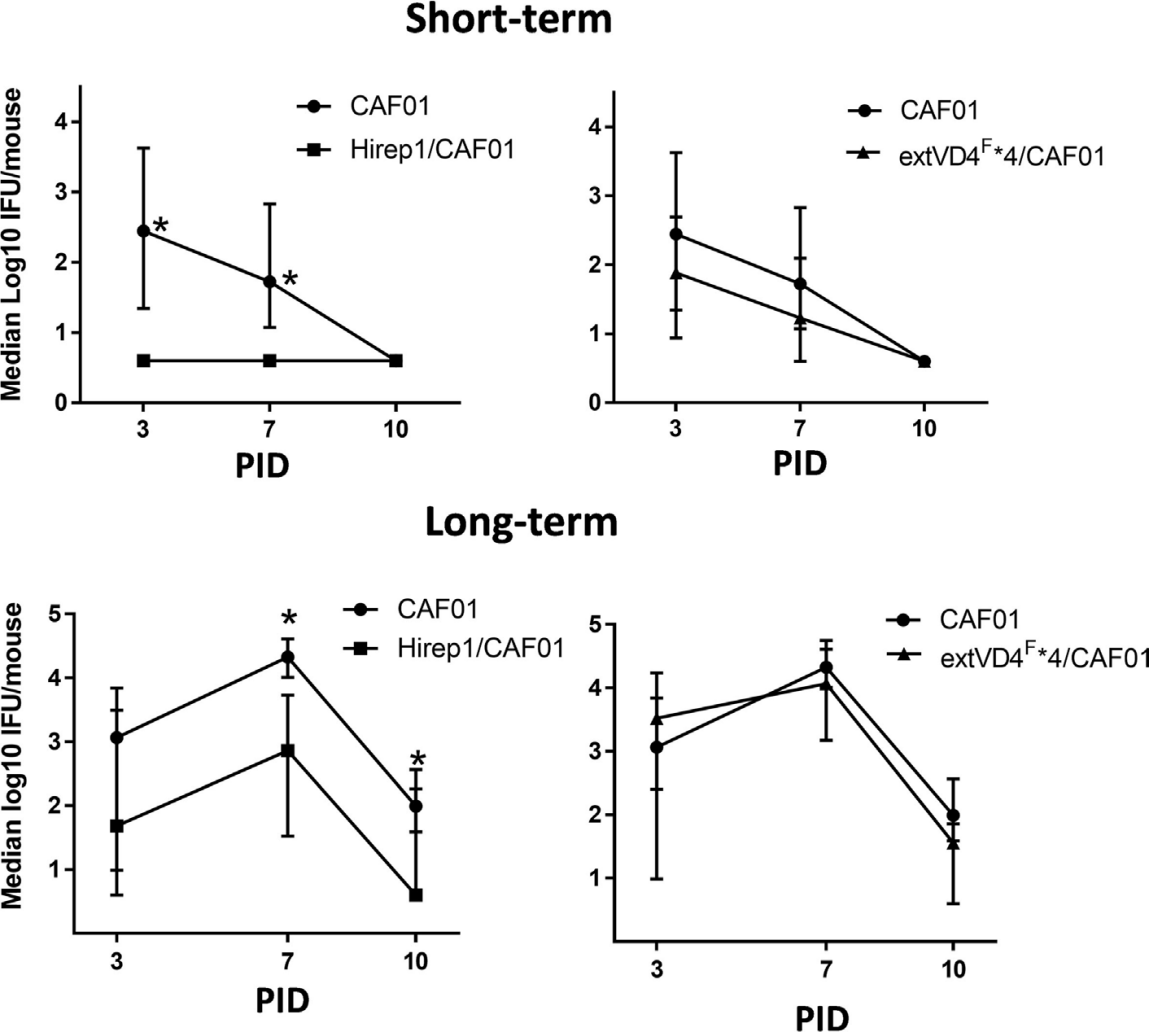
Short- and long-term protection after heterologous immuno-repeat 1 (Hirep1) and extended VD4’s from serovar (Sv) F (extVD4^F^*4) vaccination. B6C3F1 mice were vaccinated three times with Hirep1 or extVD4^F^*4 in CAF01 simultaneously by the subcutaneous and intranasal routes. 6 weeks (*n* = 8, short-term) or 72 weeks (*n* = 10–12, long-term) post-vaccination the mice were challenged with 1 × 10^6^ IFU or 4 × 10^3^ IFU of *Chlamydia trachomatis* (*C.t*.) SvF, respectively. The infection dose in the aged mice was adjusted to 4 × 10^3^ IFU/mouse due to a much higher susceptibility to a *C.t*. infection in those mice compared to younger mice (unpublished observations). Each dot represents the median number ± interquartile range of IFU recovered from vaginal swabs at days 3, 7, and 10 post-infection (*n* = 8). The Dunn’s multiple comparison tests were used for comparison among groups. *P < 0.05 and **P < 0.01.

Based on the identical levels of T cell responses but the markedly different capacity to induce neutralizing antibodies, we continued by investigating how this would affect short- (6 weeks) and long-term (>1 year) protection against a SvF infection (Figure 4). Mice were vaccinated with either Hirep1/CAF01, extVD4F*4/CAF01, or CAF01 alone and challenged with 1 × 10^6^ IFU/mouse 6 weeks after vaccination and with 4 × 10^3^ IFU/mouse following 1 year of resting. Viable *C.t*. shedding was measured by vaginal swabbing 3, 7, and 10 days post-inoculation (PID). Hirep1 vaccinated mice were significantly protected 6 weeks following vaccination and despite a 10-fold reduction in serum IgG levels (results not shown) they sustained protective immunity 1 year after vaccination. ExtVD4^F^*4 vaccination failed to protect at both time-points post-vaccination demonstrating a key role for neutralizing antibodies in protection as well as the limitations of a CMI response on its own.

### Passive Immunization of Rag1 KO Mice with Hirep1 Antibodies

**Figure 5:**
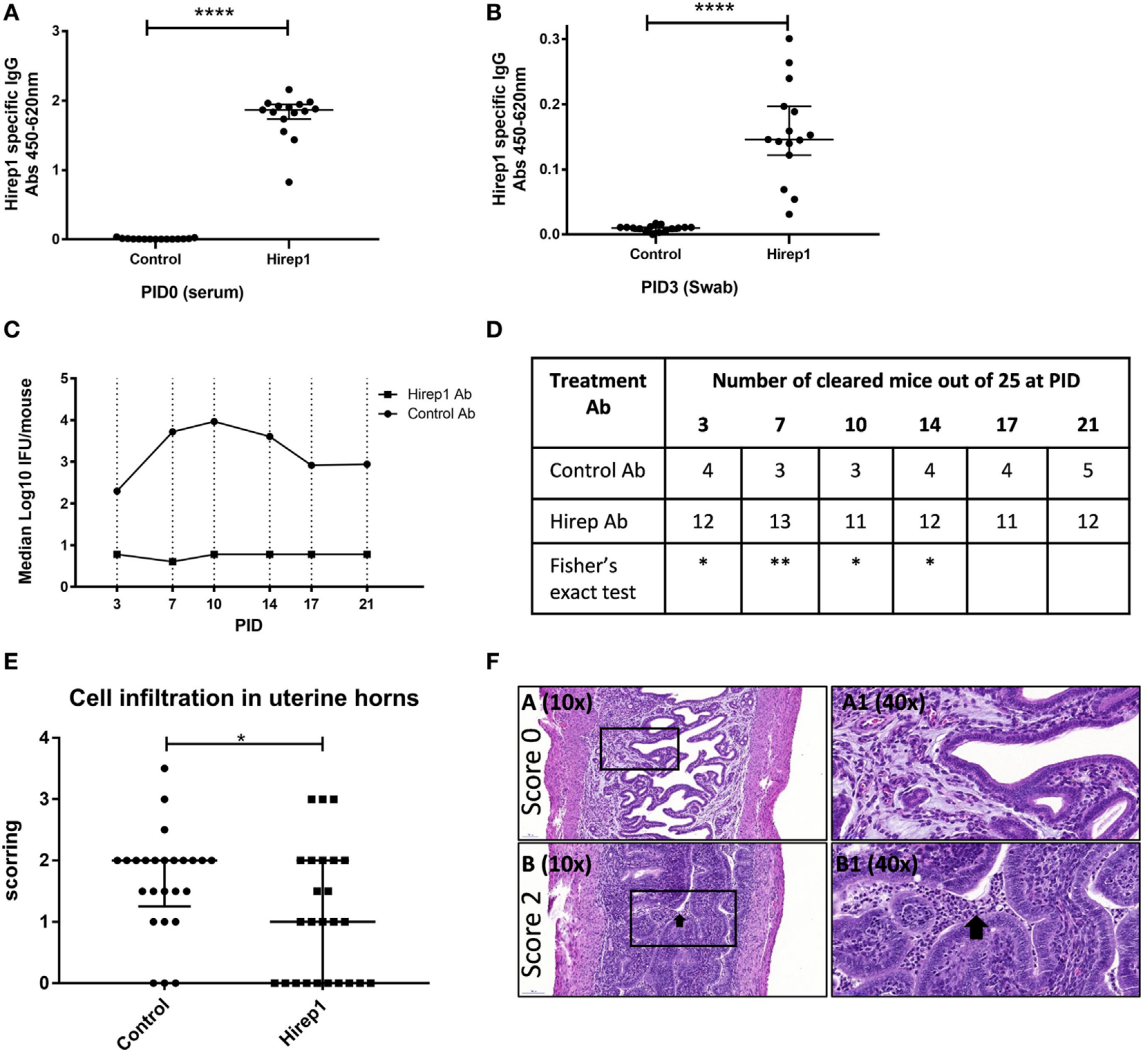
The protective role of heterologous immuno-repeat 1 (Hirep1)-specific antibodies in Rag1 knockout (KO) mice. Hirep1 vaccinated and control mice were bled, and sera were isolated, heat-inactivated, sterile filtered, and transferred to naive recipient Rag1 KO mice at day-3 of infection. Three days after transfer the mice were challenged with 4 × 10^4^ IFU of *Chlamydia trachomatis* (*C.t*.) serovar (Sv) D. **(A)** Hirep1-specific IgG in recipient Rag1 KO mice. At PID0 blood samples were drawn from 15 individual Rag1 KO mice and the level of Hirep1-specific IgG was measured in serum diluted 1:500. Each dot represents the titer in the individual mice and the median value ± interquartile range is indicated. The Mann–Whitney test was used for comparison among groups ****P < 0.0001. **(B)** Hirep1-specific IgG in post-inoculation day (PID) 3 vaginal swabs. Each dot represents the titer in the individual 15 mice and the line indicates the median ± interquartile range. The Mann–Whitney test was used for comparison among groups ****P < 0.0001. **(C)** Median log 10 IFU/mouse during the infection period. Each dot represents the median number of *C.t*. IFU recovered from vaginal swabs at days 3, 7, 10, 14, 17, and 21 post-infection. The results represent a pool of two individual experiments (*n* = 25). **(D)** Number of cleared mice out of 25 at the individual time-points post-infection. Fisher’s exact test was used for comparison, *P < 0.05. **P < 0.01. **(E)** Following euthanasia on PID21, the genital tracts were removed en bloc and processed (hematoxylin and eosin staining) for histopathologic evaluation. The genital tract tissues were evaluated for the degree of inflammatory infiltrates/presence of inflammatory cells according to a semi-quantitative scoring system from 0 to 4 (see Materials and Methods). Each dot represents the individual scoring. The median score ± interquartile range is indicated. The Mann–Whitney test was used for comparison among groups *P < 0.05. **(F)** Representative section of a genital tract with a score 0 referring to no cellular infiltration (top panels). Representative section of a genital tract with a score 2, referring to confluent cellular infiltration and furthermore luminal exudate with inflammatory cells (black arrow) (lower panels).

As all these investigations pointed to a dominant role for neutralizing antibodies in the protection promoted by the Hirep1 vaccine, we continued by characterizing the role of Abs during a primary infection independent of T and B cells. We transferred Hirep1-specific serum to Rag1 KO mice producing no mature T or B cells. We performed two individual experiments. B6C3F1 mice were vaccinated SC three times with Hirep1/CAF01 or CAF01 alone. The sera were isolated from vaccinated mice, heat-inactivated, sterile-filtered and transferred to Rag1 KO mice (2.7 donor:1 recipient) 3 days before infection. At PID 0 the Rag1 KO were bled and levels of Hirep1-specific antibodies in the periphery (serum) were measured by ELISA (Figure 5A). The mice were infected with 4 × 10^4^ IFU of *C.t*. SvD. At PID3, Hirep1-specific antibodies in the genital tract were analyzed by swabbing to determine the level of Ab transudation to the genital tract (Figure 5B). We detected Hirep1-specific antibodies in swab material from all recipient Rag1 KO mice. IFU levels were determined at PID3, 7, 10, 14, 17, and 21 and presented as a pool of the two experiments (Figure 5C). IFU levels from the individual experiments are shown in Figure S1 in Supplementary Material. The Hirep1-specific serum transfer protected Rag1 KO mice very efficiently throughout the experiment. At the level of individual animals, 12 out of 25 (48%) mice in the Hirep1 Ab-treated group completely prevented establishment of infection (non-infected at PID3), compared to 4 out of 25 (16%) in the group that received control serum (*P* < 0.05 by Fisher’s exact test), and this level was sustained throughout the infection period (Figure 5D). We finally did histopathology examinations of the genital tracts 21 days post-infection. The genital tract tissues were evaluated for the degree of inflammatory infiltrates and presence of inflammatory cells according to a semi-quantitative scoring system from 0 to 4 (Figure 5E and see Materials and Methods). The majority of the control group had confluent diffuse infiltration with inflammatory cells and some animals also luminal exudate with inflammatory cells. The median score of the control group was 2 (Figures 5E,F, lower panel). The mice from the Hirep1 group that were cleared at PID3 showed no infiltration with a score of 0 (Figures 5E,F, top panel), whereas the mice that were not cleared at PID3 showed histopathology comparable to the control group. The median score in the Hirep1 group was 1 (Figure 5E). In general, no inflammation was detected in fallopian tubes or ovarian bursa and only mild and scattered inflammatory infiltrates were seen in the vagina.

## Discussion

In the present study, we have dissected the role of a neutralizing Ab response against *C.t*. infection without the confounding influence of CMI responses. Designing experimental vaccines based on either recombinant MOMP, MOMP peptides, or MOMP DNA has been highly challenging and with limited success (31–33). Recently, we designed a recombinant engineered Hirep1 molecule [Table 1 and (20)]. Hirep1 includes T and B cell epitopes from extended VD4 regions of the most prevalent SvD–F (extVD4s) including the species-specific neutralizing LNPTIAG epitope (34, 35).

Here, we describe a comparative evaluation of Hirep1 and a construct based on a homologous repeat of the extVD4^F^*4. This molecule showed overall similar immunogenicity but lacked the ability to induce neutralizing antibodies. Despite the high degree of sequence similarities, the extVD4^F^*4 generated immune serum recognized predominantly a SvF-specific region RIAQPR and had markedly different specificity against the conserved LNPTIAG region previously shown to contain neutralizing epitopes for SvF (34). In contrast, Hirep1 generated sera recognized a much broader epitope repertoire covering both the conserved and serotype-specific regions confirming previously published results (20). The functional Ab response, in terms of neutralization, was blocked with a peptide covering FDTTTLNPTIAGAGDVK demonstrating that although Hirep1 immunization induced antibodies that recognized different parts of the VD4 region only the LNPTI region was a target for neutralizing antibodies. Thus, although LNPTI is conserved between extVD4^F^*4 and Hirep1, subtle changes in aa surrounding this region have marked effect on the specificity of the Ab response—a challenge for vaccine design that the Hirep1 vaccine molecule addresses. Previous studies using MOMP have also shown high sensitivity of amino-acid changes on the neutralizing Ab response (36). Kari et al. immunized cynomolgus macaques with native MOMP purified from the clinical SvA isolate A2497, which generated highly neutralizing antibodies against the homologous strain but failed to neutralize SvA HAR-13, a strain differing by only four amino acids (37).

Besides broad Sv coverage, a novel *Chlamydia* vaccine will need to induce long-lived protection, at least covering the age group of 15–29 where the infection is most prominent. Here, we show that immunization with Hirep1 formulated in CAF01 sustained protective immunity for more than 1 year, confirming several previous observations of the strong and long-lived immunity induced by the adjuvant CAF01 in both animal models (30, 38) and human clinical trials (39). Following vaccination and challenge with *C.t*. SvF, only Hirep1 was able to induce both short- and long-term protection. Thus, although extVD4^F^*4 induced a very similar CMI response, this was on its own not enough to significantly control infection, while the combined effect of neutralizing antibodies and CMI efficiently reduced the bacterial levels. The exact mechanism behind this interplay between CMI and neutralizing antibodies is unknown, but several studies have previously suggested mechanisms such as ADCC, Fc mediated enhanced phagocytosis and microbial killing (13, 14).

Despite a vast number of *in vitro* studies documenting a role of antibodies in both neutralization and complement activation (18, 40–44) the demonstration of a direct correlation between neutralizing antibodies and protection during a primary *C.t*. infection has been challenging. In general, studies in mice demonstrating a role for antibodies have predominantly demonstrated an important role during the secondary infection (in preconditioned tissue) (14, 45, 46) and no isolated role of antibodies has been suggested in those studies. In the current study, we demonstrate a protective efficacy of Hirep1-specific antibodies by transferring them to Rag1 KO mice producing no mature T and B cells (22). 48% (12/25) of mice receiving Hirep1 immune sera were uninfected day 3, and 10 of these mice had no sign of pathological changes, indicating that these mice completely controlled infection. These data strongly suggest a role for antibodies on their own in neutralizing the bacteria. The exact mechanism is not clear, but we believe that Hirep1-specific Ab molecules accumulate on the surface of *C.t*. and either directly inhibit them from binding and infecting the epithelial target cells or activate the complement system leading to direct lysis of the *C.t*. membrane. Possibly, antibodies could also slow down or even immobilize incoming bacteria *via* Ab-bacteria binding to mucin fibers that constitute Cervical-vaginal Mucus, a mechanism recently described for protection against HIV (47, 48).

The reason for the observed disagreement between our findings and previous work is most likely to be found in the level and functionality of antibodies. Previous studies have primarily investigated infection promoted antibodies (46), which in our hands have limited neutralizing capacity compared to Hirep1-specific antibodies (unpublished results). In support of the isolated capacity of antibodies to control infection are the observations by Cotter et al. demonstrating that MAbs delivered into serum and vaginal secretions of naive mice by using a backpack hybridoma system can reduce pathology (49). Similarly, studies from other animal models, have pointed to an isolated role of antibodies in protection against a primary *C.t*. infection (50).

In summary, we show that a vaccine inducing both neutralizing antibodies and CMI can significantly protect against infection in mice both short-and long-term post-vaccination. Importantly, we provide evidence that antibodies on their own can prevent the establishment of *C.t*. infection in Rag1 KO mice. This emphasizes a previously unrecognized role of antibodies as a first line of defense against *C.t*. infection and supports the inclusion of neutralizing targets in the development of future Chlamydia vaccines.

## Ethics Statement

Animal experiments were conducted in accordance with regulations of the Danish Ministry of Justice and animal protection committees by Danish Animal Experiments Inspectorate Permit 2013-15-2934-00978 and in compliance with EU Directive 2010/63 and the US Association for Laboratory Animal Care recommendations for the care and use of laboratory animals.

## Author Contributions

AO planned the study, performed the experiments, and made the laboratory analysis, statistics, interpreted data, and drafted the figures and manuscript. FF and PA planned the study, interpreted data, and revised figures and the manuscript. EL performed the histopathology examinations and revised figures and the manuscript. IR produced the recombinant constructs and revised figures and manuscript. All the authors approved the final manuscript.

## Conflict of Interest Statement

The authors declare that the research was conducted in the absence of any commercial or financial relationships that could be construed as a potential conflict of interest. PA, AO, IR, and FF are coinventors on a patent application relating to *C.t*. vaccines. All rights have been assigned to Statens Serum Institut, a Danish not-for-profit governmental institute.

## Abbreviations

*C.t*., *Chlamydia trachomatis*; Sv, serovar; ELISA, enzyme-linked immunosorbent assay; IFN-γ, interferon gamma; IFU, inclusion forming unit; IL-17, interleukin 17; TNF-α, tumor necrosis factor-alpha; IL-2, interleukin 2; SC, subcutaneous; IN, intranasal; FACS, flow cytometry; EB, elementary body.
