# Supplementary material for: Protective Effect of Vaccine Promoted Neutralizing Antibodies against the Intracellular Pathogen Chlamydia trachomatis
Source: Front Immunol. 2017 Dec 11;8:1652. doi: 10.3389/fimmu.2017.01652 (PMC5732375; doi:10.3389/fimmu.2017.01652)
Supplement: Supplementary file 1 [file image_1.pdf]

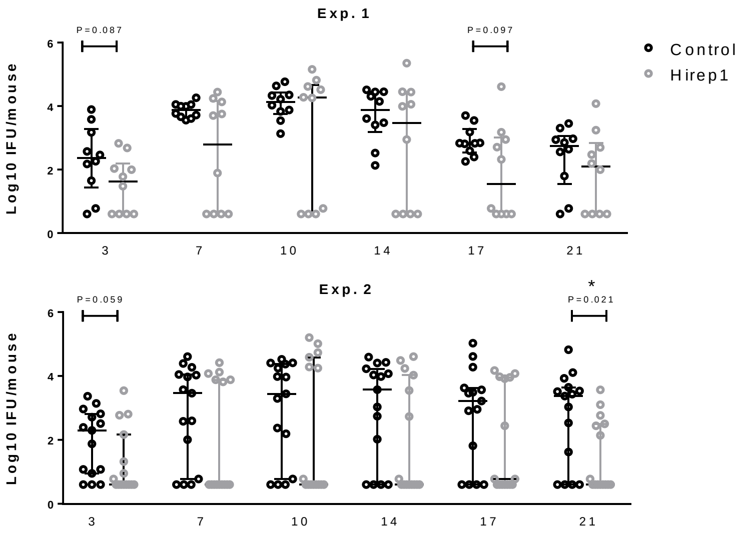

Supplementary Figure S1. Protection in Rag1 KO mice receiving Hirep1 specific serum or control serum. Two individual experiments were performed. Each dot represents the log10 number of *Chlamydia* IFU recovered from vaginal swabs at day 3, 7, 10, 14, 17 and 21 post infection. Median log10 IFU/mouse ± interquartile range are indicated. The Mann-Whitney test was used for comparison among groups *, $P<0.05$
